# Impact of Indoor Physical Environment on Learning Efficiency in Different Types of Tasks: A 3 × 4 × 3 Full Factorial Design Analysis

**DOI:** 10.3390/ijerph15061256

**Published:** 2018-06-13

**Authors:** Lilin Xiong, Xiao Huang, Jie Li, Peng Mao, Xiang Wang, Rubing Wang, Meng Tang

**Affiliations:** 1 School of Public Health, Southeast University, Nanjing 210003, China; hzxionglilin@163.com; 2 Department of Environmental Health, Nanjing Municipal Center for Disease Control and Prevention, Nanjing 210003, China; 3 Department of Hygiene, School of Public Health, Xiangnan University, Chenzhou 423000, China; huangxiao1998@163.com; 4 School of Civil Engineering, Shenzhen University, Shenzhen 518060, China; 15250986656@163.com; 5 Department of Construction Management, School of Civil Engineering, Nanjing Forestry University, Nanjing 210037, China; shadowsmyth@163.com (P.M.); yzwx97@163.com (X.W.); wangrubinghappy@163.com (R.W.); 6 Jiangsu Key Laboratory for Biomaterials and Devices, Southeast University, Nanjing 210009, China

**Keywords:** learning efficiency, task type, indoor physical environment, environmental factor, full factorial design

## Abstract

Indoor physical environments appear to influence learning efficiency nowadays. For improvement in learning efficiency, environmental scenarios need to be designed when occupants engage in different learning tasks. However, how learning efficiency is affected by indoor physical environment based on task types are still not well understood. The present study aims to explore the impacts of three physical environmental factors (i.e., temperature, noise, and illuminance) on learning efficiency according to different types of tasks, including perception, memory, problem-solving, and attention-oriented tasks. A 3 × 4 × 3 full factorial design experiment was employed in a university classroom with 10 subjects recruited. Environmental scenarios were generated based on different levels of temperature (17 °C, 22 °C, and 27 °C), noise (40 dB(A), 50 dB(A), 60 dB(A), and 70 dB(A)) and illuminance (60 lx, 300 lx, and 2200 lx). Accuracy rate (AC), reaction time (RT), and the final performance indicator (PI) were used to quantify learning efficiency. The results showed ambient temperature, noise, and illuminance exerted significant main effect on learning efficiency based on four task types. Significant concurrent effects of the three factors on final learning efficiency was found in all tasks except problem-solving-oriented task. The optimal environmental scenarios for top learning efficiency were further identified under different environmental interactions. The highest learning efficiency came in thermoneutral, relatively quiet, and bright conditions in perception-oriented task. Subjects performed best under warm, relatively quiet, and moderately light exposure when recalling images in the memory-oriented task. Learning efficiency peaked to maxima in thermoneutral, fairly quiet, and moderately light environment in problem-solving process while in cool, fairly quiet and bright environment with regard to attention-oriented task. The study provides guidance for building users to conduct effective environmental intervention with simultaneous controls of ambient temperature, noise, and illuminance. It contributes to creating the most suitable indoor physical environment for improving occupants learning efficiency according to different task types. The findings could further supplement the present indoor environment-related standards or norms with providing empirical reference on environmental interactions.

## 1. Introduction

The prevalence of sick building syndrome (SBS) symptoms leads to increasing concern about indoor environment in these days, because occupants spend most of their time learning or working indoors [1,2,3,4]. It was researched that indoor physical environment was significantly correlated to office work productivity [2,5,6]. A comfortable physical environment may stimulate work efficiency or help occupants remain productivity while environmental discomfort may result in negative productivity [7,8,9]. Effects of physical learning environment especially on cognitive load can be regarded as a determinant of the effectiveness of instruction [10]. In reviewing prior literature, temperature, noise, and illuminance are found the most common factors which influence occupant task performance or learning efficiency. For instance, Lan et al. (2011) investigated the effects of thermal discomfort on health as well as the performance of tasks simulating office work, and found that the subjects took lower work performance while warm (30 °C) than in thermoneutral condition (22 °C) [11]. To some extent, this is consistent with Seppanen and Fisk’s (2005) research, which revealed that work performance might be improved within the room air temperature of 20~23 °C [12]. Imhof (2014) showed that distracting and irrelevant sound interfered with information processing efficiency, which was in line with other findings [13,14,15,16]. Impact of stimulus background illumination seems to be another variable that influences visual perception, cognitive processing, and behavioral responses [17,18].

To our best knowledge, there was some empirical support for interactions of physical environmental factors on human comfort or task performance [19,20,21]. Most of the papers focused on the combinations between temperature and illuminance, followed by those between temperature and noise [22,23,24,25], but the research on how learning efficiency is affected by the concurrent multitude of ambient factors is still limited. Although there was support for interactions among noise, heat, and illuminance on cognitive performance [26], which interactive environmental scenario where the performance comes to the top was still not well understood. Besides, occupants always engage in different learning or working tasks indoors, most of which are related to perceptual processing, memory processing, problem-solving, or attentional focusing [21,27]. It appears that there may be fairly obvious disparities among learning efficiency in different tasks if ambient temperature, noise, and illuminance affected it simultaneously. However, there is little published work finding such environmental combined effect on learning efficiency based on task types. It is envisaged that the optimized physical environment should be created depending on both environmental interactions and learning tasks, but relevant information is currently unavailable.

We hypothesized that the indoor physical environmental factors, viz. temperature, noise, and illuminance, exerted significant main effect and concurrent effect on learning efficiency according to task types, and different interactive environments could be found when the efficiency peaked to maxima in different tasks. The objective of this study is to explore the impacts of indoor physical ambient factors including temperature, noise, and illuminance and their interactions on learning efficiency (viz. accuracy rate (AC), reaction time (RT), and performance indicator (PI)) in different types of learning tasks (i.e., perception, memory, problem-solving, and attention-oriented tasks). Furthermore, it aims to identify the optimal interactive environments where the highest learning efficiency achieved in different types of tasks with considering simultaneous effects of three factors. The paper provides guidance for building users to conduct effective environmental intervention by controlling environment indoors. It puts emphasis on creating the optimal physical environment indoors, with considering concurrent effect of the multitude of ambient factors, to help occupants achieve the highest learning efficiency in different types of tasks. Moreover, it provides a reference regarding environmental interactions for the present indoor environment-related standards or norms, which is conductive to promoting the human-centered design of buildings further.

## 2. Material and Methods

### 2.1. Environmental Design

The full factorial experiment was carried out in an environment-controlled university classroom (11.7 m × 9.0 m × 3.0 m), which had been used for more than five years. Ten individual desks were previously arranged in five rows in the center of it. Natural light indoors was provided through single-sided windows, over which were hung the movable shade curtains. A complete design led to 36 scenarios, with three main environmental factors including temperature at three levels, noise at four levels, and illuminance at three levels. Design for environmental scenarios in the 3 × 4 × 3 full factorial experiment is shown in Table 1. Criteria for factor levels selected are illustrated.

Limited to the experiment conducted in October and November in 2016, the temperature indoors was maintained at three intended levels of 17 °C, 22 °C, and 27 °C by controlling central air-conditionings. These temperature levels, ranging from moderately cool to moderately warm, were identified within the acceptable range of indoor temperatures in such a season after a pilot study. Simultaneously, they were within the operative range set by air-conditionings, bringing much convenience to create different environmental conditions. The lowest temperature, 17 °C, represented the moderately cool exposure found in the pilot study while the highest temperature, 27 °C, represented the moderately warm exposure. The temperature of 22 °C, at the mid-point of the intended range, was selected because it was considered to create a condition for optimal performance [28]. Temperatures were measured using a U.S. Gray Wolf indoor air detector (GrayWolf Sensing Solutions, LLC, Shelton, CT, USA). Three kinds of thermal environments were defined according to the actual measurements, including cool environment (17.32 ± 0.47 °C), thermoneutral environment (22.21 ± 0.63 °C), and warm environment (27.11 ± 0.68 °C).

Noises indoors are found in a pilot study that they are commonly composed of talking noises, air-conditioning noises, distant outside car horns, and even shoes clattering. It is therefore the noise distractor in experiments was previously recorded in the classroom to simulate the actual acoustic environment for daily in-class learning. Ambient noise in the experimental classroom was 40 dB(A) when it was unoccupied, similar to the acoustic condition in daily ones. 50–80 dB(A) were estimated by Mackenzie as typical of indoor noises [29], but it was only measured with occasional peaks to 80 dB(A) in the pilot study. Therefore, the noises of 40 dB(A), 50 dB(A), 60 dB(A), and 70 dB(A) were finally selected as variables in actual experiments. To ensure the noise levels as homogeneous as possible inside, they were controlled by four multimedia players which were positioned in four separate corners. A noise dosimeter, model AWA5610D (Hangzhou Aihua Instruments Co. Ltd., Hangzhou, China), was used for measurement. During the experiments, all the windows and doors were closed to minimize external distractions. There were four different acoustic environments finally identified: fairly quiet environment (40.85 dB(A) ± 0.43 dB(A)), relatively quiet environment (51.73 dB(A) ± 0.55 dB(A)), relatively noisy environment (61.83 dB(A) ± 1.20 dB(A)), and fairly noisy environment (72.32 dB(A) ± 0.74 dB(A)).

Natural illuminance was measured indoors on a sunny day in the pilot study (no fluorescent lights turned on). Three levels were employed at 50–70 lx, 200–400 lx, and 2000–2400 lx according to distance from single-sided windows. For better variable controls, mid-points of these three ranges, 60 lx, 300 lx, and 2200 lx, were finally selected based on Standards for Lighting Design of Buildings (GB50034-2013) and the previous research work [30]. 60 lx is lower than the minimum standard for indoor lighting (100 lx), 300 lx is the recommended illuminance for libraries or classrooms, and 2200 lx far exceeds indoor illuminance comfort levels. Lighting environment in experiments was mainly created by natural light. Curtains were closed for shading when the illuminance indoors was further higher than what was needed. Diminutive cool-white trichromatic lamps were additionally arranged on the top left corner of each desk for ideal illuminance controls. To make the illuminance as homogenous as possible all around the subjects, lamps were designed adjustable. Therefore, illuminance on desks could be controlled flexibly based on the actual measurement. All the lamps were previously tested to avoid risks of spontaneous uncontrollable flicker. Illuminance was measured using an illumination photometer, model TES-1332A (TES Electrical Electronic Corp., Taibei, China), with three lighting scenarios differently classified, i.e., dark environment (61.55 ± 1.79 lx), moderately light environment (319.75 ± 13.89 lx), and bright environment (2202.14 ± 22.21 lx). Experiments started only if the illuminance on all desks met experiment requirements.

All instruments were calibrated before each experiment. Layouts of measurement on temperature, noise and illuminance were designed according to Methods of Health Monitoring for Public Places (GB/T 18204.1-2013), especially the section regarding physical factors. All other physical environmental factors were held at a fixed level controlled by central air-conditionings, and thus, they could be kept relatively constant in the whole experimental course. The series of experiments lasted 36 days in total, with one scenario per day in the sequence that is previously set on basis of level changes of each factor. All scenarios experimented were independent with each other. 

### 2.2. Subjects

Subjects were required to participate in the whole course of the experiment with everyone attending all 36 environmental scenarios. Considering the long experiment period, 10 college students (five males and five females) aged between 20 and 24 years old were recruited as voluntary subjects from a previous large-scale ground survey based on the following criteria: non-smokers or alcoholics, absence of diseases, and normal or corrected-to-normal version. All subjects recruited were required to have more than three years of local experience to avoid the effects of environmental inadaptability [31,32]. The information was obtained from questionnaires distributed during the recruitment survey. Besides, none of them was examined medically.

To minimize individual differences, the subjects were required to receive enough sleep and a regular diet and keep emotional stability in experiment period. Activities probably causing experimental bias were also limited as follows. Drugs were not allowed on experimental days. Alcohol could not be consumed within 12 hours before each exposure. Teas or other drinks that function as stimulating nerves and cardiovascular systems were not allowed within eight hours before each exposure. Strenuous exercises were also not allowed within three hours before each exposure, which helped stabilize blood sugar and avoid neural hyper excitability. Subjects were instructed to wear long-sleeved cotton T-shirts, long trousers, long underclothes on the top and bottom, socks, and shoes (1.0 clo), corresponding to the typical clothing indoors based on local climates. To avoid distractions from subjective evaluations on indoor physical environments, environmental variables designed were introduced to subjects using blind intervention approach.

### 2.3. Learning Tasks

Occupants mainly engage in brainwork through a series of cognitive activities regarding information processing (receiving, explaining, organizing, and extracting) in essence [33]. Cognition, as the whole processing cycle of information, can be divided into four main categories including perception, memory, problem-solving performance, and attention [8]. According to the characteristics of brainwork and cognition, cognitive capacity can be roughly regarded as an indicator of learning efficiency. Therefore, on basis of reliability, validity, difficulty, and discriminability separately, four classical tests representing each category of cognitions were selected from both domestic and international achievements in the existing psychological domain, and were conducted to measure the efficiency of different types of learning tasks in this study (Table 2). Detailed information about each task is following.

#### 2.3.1. Perception-Oriented Task

The Rochester color word test was conducted to test the capacity of virtual perception [34]. A total of 15 words of colors in another color were presented on papers. Subjects were asked to pick out the word itself or its color sequentially. 

#### 2.3.2. Memory-Oriented Task

Recognition of meaningless images was conducted to test capacity for short-term memory [35]. A total of 10 meaningless images on paper were presented for only 10 s. When time was up, the subjects were asked to pick them out from all 20 meaningless images on another paper as quickly as possible. 

#### 2.3.3. Problem-Solving-Oriented Task

Reading comprehension was conducted to test problem-solving performance [36]. Subjects were asked to pick out the only correct answer from multiple choices based on their own understandings. Previously printed out, five independent questions were randomly distributed to each subject from the administrative ability tasks for national civil servant selections. 

#### 2.3.4. Attention-Oriented Task

Number searching test was conducted to test attention [37,38,39]. Numbers 0 through 99 were sequenced out of order on papers. Subjects were asked to search 15 designated numbers in normal order from these 100 numbers. 

The output of work is related to both quantity and quality. To evaluate learning efficiency in different tasks, accuracy rate (AC), reaction time (RT), and the final performance indicator (PI) were chosen as three dependent variables [8,36,40,41,42], where AC is the percentage of correct answers and RT is the time for which the task was completed, so that PI represents the overall rate correctly completed in each task. The relationship among them is as follows:

PI=AC/RT

To simulate the most common routine performance, subjects were singly exposed without anyone under close supervision. The completion time of each task was recorded by subjects themselves while the percentage correct and the final PI were then calculated by research associates after experiments. During the week prior to the formal experiment, subjects were given one introduction session and two other training sessions to make themselves fully familiarized with the task procedure as well as the task rules, so that the potential deviations caused by different extent of familiarity with tasks was cautiously avoided. All answers should be given in pens.

**Figure 1:**
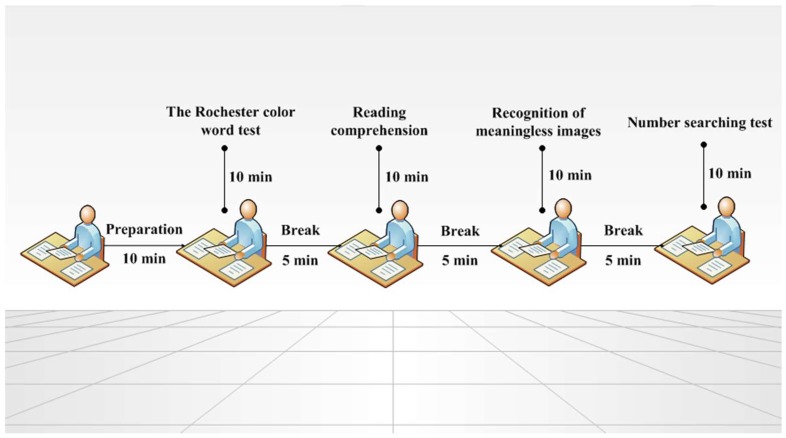
Experiment schedule in each environmental scenario.

Each test lasted around 10 min without putting too much intellectual pressure on subjects. Besides, a five-minute break was set between each test with the next. Therefore, impact of fatigue in the long task duration could be ignored. Subjects were asked to arrive 10 min before the first task starting, in which, they were instructed to sit on their separate seats in a designated classroom and were only allowed to do some quiet activities such as reading, listening to music, and doing homework. Therefore, all 10 subjects exposed for around one hour at each time, starting at approx. 2.00 p.m. All tests conducted under every scenario were independent with each other without order effect. The experiment schedule is designed as shown in Figure 1, for much quality control and experimental efficiency in the whole process.

### 2.4. Data Analysis

Factor levels of the physical indoor environment (i.e., temperature, noise, and illuminance) shown in mean ± standard deviation (SD), were measured within their individual 95% confidence interval. MANOVA in full factorial model was then conducted to explore the main effects and interaction effects of the three environmental factors on learning efficiency in different tasks. Data collected was tested for normal distribution with using the Kolmogorov-Smirnov Test. Non-normal distributed data were computed with statistical disposal after a log-normal transformation. All analyses were performed using SPSS version 22.0 (IBM, Armonk, NY, USA). Significance was defined as *p* < 0.05.

## 3. Results

### 3.1. Main Effects of Indoor Physical Environment on Learning Efficiency in Different Tasks

At the protected significance level, the main effect of temperature on AC was significant, which was found only in The Rochester color word test. That means no significance changed with respect to impact of temperature on perception AC when noise or illuminance increased or decreased. It seems temperature, noise, and illuminance failed to affect AC individually in three other tasks. We detected significant main effects of illuminance on RT and PI regarding visual perception (RT: F = 13.306, *p* < 0.05; PI: F = 13.286, *p* < 0.05). It is shown that RT and PI across perception, which were individually influenced by illuminance, changed slightly as temperatures from 17 °C to 27 °C and noise levels from 40 dB(A) to 70 dB(A). Therefore, for the perception-oriented task, temperature indoors was regarded as a dominant factor affecting AC while illuminance was another dominant factor that influenced both RT and PI.

Revealed from the results, temperature, noise and illuminance failed to significantly affect AC in the memory-oriented task, the individual effect on AC among which was found to be sensitive to the two others (*p* > 0.05, n.s.). On the contrary, there was significant main effect of each of these three factors on RT and PI in recognition of meaningless images (*p* < 0.05).

Both temperature and noise exerted significant main influence on RT in reading comprehension (temperature: F = 5.207, *p* < 0.05; noise: F = 14.855, *p* < 0.05), so did they on RT in number searching test (temperature: F = 4.544, *p* < 0.05; noise: F = 4.634, *p* < 0.05). That is to say, it was obvious that the time in which subjects reacted in the problem-solving-oriented and attention-oriented task changed, when indoor temperature or noise levels individually increased or decreased in the range experimented. According to the final performance of learning efficiency, however, the dominant environmental factors were quite different from each other in these two types of tasks. Significant main effects of noise and illuminance were observed on PI in problem-solving process (F = 3.277, *p* < 0.05), while significant main effect of temperature was only found regarding attention (F = 10.197, *p* < 0.05). Details of the main effects of indoor temperature, noise and illuminance on learning efficiency in these four tasks are depicted in Table 3.

### 3.2. Interaction Effects of Indoor Physical Environment on Learning Efficiency in Different Tasks

As shown in Table 4, there are different interaction effects of indoor temperature, noise and illuminance on efficiency in four learning tasks. The Rochester color word test was the only experiment, in which AC was concurrently affected by all three ambient factors. Significant interactions between temperature and noise were found on AC across perception (AC: F = 2.472, *p* < 0.05). However, it was detected in this task that the final PI was significantly influenced by noise interacting with illuminance instead of with temperature. Therefore, it should give priority to the simultaneous modulation between noise and illuminance for promoting the final perception performance. Moreover, we found perception-related RT was rather more sensitive to the multitude of environmental factors than AC and PI. There was more than one kind of significant environmental interactions on RT of perception, including the interaction between temperature and noise as well as that between noise and illuminance (temperature × noise: F = 2.155, *p* < 0.05; noise × illuminance: F = 2.623, *p* < 0.05). 

There were the most combined effects found in recognition of meaningless images despite none being found for AC. The significant interaction effect between temperature and noise was shown on RT (F = 4.703, *p* < 0.05) and PI (F = 3.888, *p* < 0.05) for short-term recollection. Meanwhile, they were both interactively influenced by temperature, noise, and illuminance together with great significance (RT: F = 3.165, *p* < 0.05; PI: F = 2.764, *p* < 0.05). Besides, it was found that RT across memory was significantly affected by the crossover between temperature and illuminance (F = 3.727, *p* < 0.05). Hence, as for memory-oriented tasks, all three factors, such as ambient temperature, noise, and illuminance, should be comprehensively taken into account when learning zones need designing.

Learning efficiency in reading comprehension was not sensitive to the concurrent effects of environmental factors compared with others. RT was the single dependent variable reflecting obvious interactions in this task, which was only significantly detected between temperature and noise (F = 4.511, *p* < 0.05). No interaction effect was observed on AC and PI in reading comprehension (*p* > 0.05, n.s.). It was thus illustrated that learning efficiency in problem solving process depends much on the individual main effects of ambient factors rather than their interactions.

As for the attention-oriented task, combined effect between noise and illuminance was indicated significantly modulating both RT (F = 2.547, *p* < 0.05) and PI (F = 2.191, *p* < 0.05). Besides, two more interactive scenarios were separately found in this task. RT was also significantly altered by interactions of the multitude of all three environmental factors (F = 1.883, *p* < 0.05) while PI was significantly affected by temperature and noise (F = 2.287, *p* < 0.05). There was still no significant interaction shown on AC in the number searching test (*p* > 0.05, n.s.).

### 3.3. Optimal Environmental Scenarios in Different Types of Learning Tasks

As PI reflects the final performance with considering both quality and quantity of learning output, the optimal environmental scenarios where the highest learning efficiency was achieved in different tasks were finally identified according to it. Table 5 illustrates results of the final PI on basis of task types among 36 full factorial designs.

There were clear disparities among the most suitable thermal conditions for efficiency, which were affected by the simultaneous temperature, noise and illuminance in different tasks. Subjects gained the peak level of learning efficiency with regards to perception and problem-solving-oriented tasks in the thermoneutral environment. Differently, cool exposure provoked the highest efficiency in the attention-oriented task while warm exposure worked regarding recollection.

Fairly noisy exposure (70 dB(A)) interacted with ambient temperature and illuminance was commonly found leading to the lowest efficiency in different tasks. On the contrary, a quieter environment was more suitable for cognitive activities in each kind of tasks. The acoustic environment, however, was not equal to the noise of zero level. It was illustrated that learning efficiency commonly peaked at maxima when there was less than 50 dB(A), and a stricter acoustic environment was required in the problem-solving-oriented and attention-oriented task than in the other two tasks.

Influenced by temperature and noise concurrently, the highest learning efficiency was achieved mostly in lighting exposure of no less than 300 lx, the illuminance level in general indoor environmental-related standards or norms. Specifically, perception or attention-oriented tasks usually depended on bright condition interacting with temperature and noise, with the highest performance appearing on level of 2200 lx. The optimal environmental scenarios based on task types are depicted in detail, shown in Table 5.

## 4. Discussion

Different combinations of environmental factors have been researched for improving work or learning performance in prior studies [19,20,21]. However, they mostly failed to give strong evidence for the simultaneous combined impact of physical environmental environment on learning efficiency according to types of tasks. To our best knowledge, this is a study which makes up for lack of the available information on how multiple physical environmental factors including temperature, noise, and illuminance, affect learning efficiency indoors concurrently in different kinds of learning tasks (viz. perception, memory, problem-solving, and attention-oriented tasks) from a cognitive perspective of the whole informational processing cycle.

Consistent with previous studies [8,36,40,41,42], we observed the final performance indicator (PI), which reflects the combined learning efficiency was significantly influenced by different levels of temperature, noise, and illuminance. Hence, the optimal scenarios where the highest PI was achieved in four different tasks, are further discussed based on their main or interacted effects in the current study.

Interactions on perception were found between visual and thermal environments [43,44]. The focus in this research is on the visual perception, an integration of objective reality that directly reflects on human sensory organs, among which, eyes are considered to be the most important bridge for information input [45,46,47]. It differs from superior cognition processes like reading comprehension or attention [46]. Therefore, the perception-oriented task highly stresses the eye-hand coordination in practical settings. According to physiological theory and cognitive psychological theory, up to 80% of the information received from outside is processed by the visual pathway, and color is regarded as the primary visual language [48]. This is because the visual stimulation from light is much stronger and more direct than functions of other external physical media [8]. Hence illuminance, which showed the exclusive main effect in this task, could be regarded as the primary factor for perceptual efficiency. As the illuminance level increased from 60 lx to 2200 lx, the PI level of the subjects correspondingly increased in the range experimented. There were also significant interactions between light and noise conditions with regard to the perception-oriented task. This accords with the earlier observations, which demonstrated interactions on self-assessed work suitability between light and noise in simulated office [49]. If an interaction is present, arousal, affect, or activation might concurrently contribute to their plausibility as mediators of cognition [50,51]. In the perception-oriented task, the perceptual processing to maxima was detected in the thermoneutral, relatively quiet and bright environment. It seems possible that bright-light exposure with mild temperature leads the subjects to high arousal and high activation when visual sensation is simulated. Supported by Tham’s (2004) study in the tropics, office workers were activated in a slightly cool work environment within the range of 20–24 °C, in which they felt more energetic [52]. Besides, in another experiment to explore interactions between noise and illuminance on perception, subjects were self-reported to perform better under low intelligibility noise based on their activated pleasure [53]. Therefore, the finding in the perception-oriented task is consistent with the arousal theory that the optimum level of performance for easy tasks shows in the region of high arousal [54,55], which could be found in earlier findings. 

As for memory-oriented tasks, different environmental interactions on memory performance may be attributed to disparities among the targets to memorize. Hygge and Knez (2001) found the significant crossover interactions between noise and temperature on the recall of the text but those between noise and light on the free recall of the emotionally toned words [23]. Whereas in the present research, the final PI on meaningless images recognition was significantly affected by concurrent temperature, noise and illuminance indoors. Similar to RAM in PC, short term memory refers to the ability to store temporary information in the brain, the limit of which ought to be acknowledged that it is susceptible when the information is reserved or processed. At the same time, ambient interference is quite disruptive to human memory with respect to visually presented items [56]. Therefore, the recall of images seems to be more sensitive than that of text or tuned words to temperature, noise, and illuminance surroundings, leading to a much more sophisticated combined effect. The highest efficiency for recollection was achieved in warm, relatively quiet, and moderately light environments. In other words, when warm air with a bit of noise was needed in moderate-lighting exposure, the ability to store and process temporary information was much more promoted in human brain. Findings that impaired recall with heat at the high noise level but improved recall at the low noise level were illustrated in the current research, which is not in accordance with the prior result of noise increasing arousal or activation, and mild heat decreasing it [23]. Hence, it is likely that the noise increasing arousal model was not thoroughly suitable for the memory-oriented task. Increased activation on recollection efficiency caused by lower noise levels in warm and moderately lighting condition, the finding of which corroborates to the earlier theoretical conjecture to some extent, showing that ambient factors might act directly on cognitive performance without any arousal, affect or activation as mediators [57]. Furthermore, the optimal environment regarding memory in this study is some inconsistent with that in the research of Park et al. (2013), who found a higher illuminance level (700 lx) was more beneficial to the efficacy of short term memory than the low one because it significantly helped alleviate mental load in retention period [58]. However, the factorial design in their experiment only focused on the effect of illumination condition on memory without temperature or noise into account. Thus, it could be illustrated from ours that the increased illuminance level was counteracted by low noise with heat and moderate light. 

Subjects achieved the highest learning efficiency under thermoneutral and moderate-lighting exposure with the lowest noise in the problem-solving-oriented task. However, none significant combined effect on problem-solving efficiency was found among ambient temperature, noise and illuminance indoors, which was quite contrary to our previous hypothesis that interactions could be significantly found in each type of task. Therefore, in terms of environmental controls in learning zones where much brainwork is needed, main effect rather than interactions of ambient factors could be primarily emphasized for efficiency. Noise is suggested as a dominant factor due to its individual main effect. As the noise level increased from 40 dB(A) to 70 dB(A), the final PI level of the subjects adversely decreased in the range experimented. The reason is probably that when noise levels are raised, people’s central neutral systems are easily stimulated by surroundings, leading to a decline in their cognitive functions [59,60]. Compared with the perception and memory-oriented tasks, the requirements for an acoustic environment were stricter in regards to critical thinking. This is because in the problem solving process, thinking could be an indirect reaction that the brain conducts for objectives, which is superior to a direct perceptual or retention period. Illuminance is another ambient factor that illustrates the significant main effect on problem-solving efficiency. Although the trend shows that as the illuminance level increased from 60 lx to 2200 lx, the PI level increased, it does not seem clear. If there were small disparities of learning efficiency under the illuminance of 300 lx and 2200 lx, the former level should be recommended from the energy-saving perspective, which is confirmed by the present findings that 300 lx was an illuminance level for optimal environmental efficiency in the problem-solving task.

As another aspect reflecting cognitive performance, attention refers to the ability against ambient interference when disturbed by irrelevant information. Interactions between noise and temperature found in our study could be supplemented by conclusions in the previous research that noise did not interact with heat in attention tasks [61]. This is because the levels of noise (72 dB, 80 dB, and 90 dB) and temperature (22.7~34.4 °C) selected might not quite simulate an actual indoor environment. According to the main and combined effect in this attention-oriented task, it was known the trend that as temperature increased, the final PI decreased was significantly affected by changing noise. At the high level of illuminance, the efficiency achieved in the environment simultaneously affected by noise and temperature was higher than that in one affected by temperature or noise individually. It could be therefore speculated that there was a synergetic interaction between noise (40 dB(A), 50 dB(A), 60 dB(A), 70 dB(A)) and temperature (17 °C, 22 °C, 27 °C) on attention. Additionally, the observed changes in PI provides evidence that bright exposure could modulate our attention with regard to interactions with temperature and noise. It seems that the increment in illuminance levels was assumed to increase arousal. The finding in Kaida’s (2012) study confirmed that humans often experienced bright illumination conditions which may increase cognitive performance [59]. As cool and bright exposure brought high arousal, the potential drowsiness due to fairly quiet conditions could be naturally counteracted. This is why the subjects were the most concentrated in a cool, fairly quiet and bright environment in the attention-oriented task. Only if each fixation is aimed instead of in the process of eye saccade does attention occur. It is where the perceptual span appears, the range of valuable information precisely captured under each gaze in cognitive process [62,63]. Visually perceptual span is also called sensory memory, in which information will not be admitted to the next stage of short term memory until paid attention to. Hence the design requirement for indoor physical environment where the highest learning efficiency peaked in attention-oriented task would be quite different from that in memory-oriented task.

Currently, the standard of “PD ISO/TR 15742—Ergonomics of the physical environment—Combined effects of thermal environment, air pollution, acoustics and illumination on humans” has been developed, but it is still at the ideas stage [25]. Simultaneously, ASHRAE Guideline 10P also puts emphasis on interactions among indoor environmental factors, in which most studies only investigated impact of one or two factors correlated to human comfort without considering what interaction effect the multitude of ambient factors may exert on learning efficiency from perspective of objective performance in cognitive processes [44]. They also do not identify the optimal environmental scenario for efficiency in different types of learning tasks. If the knowledge regarding environmental interactions could be incorporated in the indoor environment-related standards or norms, human-centered design of buildings should be further promoted. A deeper comprehension of interactions of indoor physical environmental factors would lead to a better understanding of their combined effect on learning efficiency according to types of tasks, which better helps efficient environmental intervention to create the optimal learning environment for the highest occupant efficiency.

To investigate the detailed interaction effect of indoor physical environment on learning efficiency according to different task types, a full factorial design experiment was carried out in the current study but there were some limitations. Regarding the long experimental period lasting 36 days, the experiment failed to take female response into consideration [64,65,66]. Sex-related differences will be considered in further cross-designed research to explore more details on interactions and crossovers in gender, with the existing physical environmental factors. Besides, the sample size was limited due to the complexity of experimental design. A larger size of samples will be selected in the following experiments. Our future work will focus on a comprehensive indoor environmental point of view with more multiple ambient factors taken into consideration to testify and expand the present research findings further.

## 5. Conclusions

Our observations provide substantial evidence that indoor physical environmental factors, including temperature, noise, and illuminance exerted significant main effects on learning efficiency in perception, memory, problem-solving, and attention-oriented tasks. There was a significant concurrent effect of three factors on learning efficiency, considering the final PI in four types of tasks except the problem-solving-oriented task. The optimal environmental scenarios where the top task learning efficiency was achieved were then identified based on PI under different interactions of ambient temperature, noise and illuminance. The highest efficiency came in thermoneutral, relatively quiet, and bright conditions with respect to the perception-oriented task. Subjects performed best under warm, relatively quiet, and moderate light exposure when recalling images. Learning efficiency of subjects peaked to maxima in thermoneutral, fairly quiet, and moderately light environments in problem-solving processes, while in cool, fairly quiet, and bright environment in the attention-oriented task. The findings in this research helps building users better understand the concurrent effect of ambient factors as well as facilitate indoor physical environments more efficiently for learning efficiency improvement. It could also supplement the present indoor environment-related standards or norms with providing an empirical reference on environmental interactions. More positive impact of the multitude of environmental factors will be accentuated in the future work. 

## Figures and Tables

**Table 1:** Design for environmental scenarios in 3 × 4 × 3 full factorial experiment. ^a^

	T_1_			T_2_			T_3_		
	I_1_	I_2_	I_3_	I_1_	I_2_	I_3_	I_1_	I_2_	I_3_
**N_1_**	T_1_N_1_I_1_	T_1_N_1_I_2_	T_1_N_1_I_3_	T_2_N_1_I_1_	T_2_N_1_I_2_	T_2_N_1_I_3_	T_3_N_1_I_1_	T_3_N_1_I_2_	T_3_N_1_I_3_
**N_2_**	T_1_N_2_I_1_	T_1_N_2_I_2_	T_1_N_2_I_3_	T_2_N_2_I_1_	T_2_N_2_I_2_	T_2_N_2_I_3_	T_3_N_2_I_1_	T_3_N_2_I_2_	T_3_N_2_I_3_
**N_3_**	T_1_N_3_I_1_	T_1_N_3_I_2_	T_1_N_1_I_3_	T_2_N_3_I_1_	T_2_N_3_I_2_	T_2_N_3_I_3_	T_3_N_3_I_1_	T_3_N_3_I_2_	T_3_N_3_I_3_
**N_4_**	T_1_N_4_I_1_	T_1_N_4_I_2_	T_1_N_4_I_3_	T_2_N_4_I_1_	T_2_N_4_I_2_	T_2_N_4_I_3_	T_3_N_4_I_1_	T_3_N_4_I_2_	T_3_N_4_I_3_

^a^ T_i_—temperature levels, in which, T_1_ represents 17 °C, T_2_ represents 22 °C, and T_3_ represents 27 °C. N_i_—noise levels, in which N_1_ represents 40 dB(A), N_2_ represents 50 dB(A), N_3_ represents 60 dB(A), and N_4_ represents 70 dB(A). I_i_—illuminance levels, in which, I_1_ represents 60 lx, I_2_ represents 300 lx, and I_3_ represents 2200 lx.

**Table 2:** Tests on learning efficiency in different types of tasks.

Task Type	Test
Perception-oriented task	The Rochester color word test
Memory-oriented task	Recognition of meaningless images
Problem-solving-oriented task	Reading comprehension
Attention-oriented task	Number searching test

**Table 3:** Main effects of indoor temperature, noise and illuminance on learning efficiency in different tasks.

			DF	SS	MS	F	*p*
Perception-oriented task	AC ^a^	Temperature	2	266.485	133.242	3.329	0.037 *
		Noise	3	140.617	46.872	1.171	0.321
		Illuminance	2	10.990	5.495	0.137	0.872
	RT ^a^	Temperature	2	4332.198	2166.099	2.478	0.085
		Noise	3	6634.407	2211.469	2.530	0.057
		Illuminance	2	23,257.991	11,628.996	13.306	0.000 *
	PI ^a^	Temperature	2	0.116	0.058	2.742	0.066
		Noise	3	0.166	0.055	2.623	0.051
		Illuminance	2	0.561	0.280	13.286	0.000 *
Memory-oriented task	AC ^a^	Temperature	2	143.889	71.944	0.376	0.687
		Noise	3	263.056	87.685	0.458	0.712
		Illuminance	2	27.222	13.611	0.071	0.931
	RT ^a^	Temperature	2	503.439	251.719	4.144	0.017 *
		Noise	3	1625.484	541.828	8.920	0.000 *
		Illuminance	2	802.265	401.326	6.607	0.002 *
	PI ^a^	Temperature	2	9.205	4.603	3.905	0.021 *
		Noise	3	19.891	6.630	5.626	0.001 *
		Illuminance	2	13.999	6.999	5.939	0.003 *
Problem-solving-oriented task	AC ^a^	Temperature	2	168.889	84.444	0.210	0.811
		Noise	3	2111.111	703.704	1.750	0.157
		Illuminance	2	1135.556	567.778	1.412	0.245
	RT ^a^	Temperature	2	11,086.794	5543.397	5.207	0.006 *
		Noise	3	47,443.360	15,814.453	14.855	0.000 *
		Illuminance	2	2820.614	1410.307	1.325	0.267
	PI ^a^	Temperature	2	0.035	0.017	0.949	0.388
		Noise	3	0.540	0.180	9.870	0.000 *
		Illuminance	2	0.120	0.060	3.277	0.039 *
Attention-oriented task	AC ^a^	Temperature	2	1.645	0.823	0.160	0.852
		Noise	3	1.037	0.346	0.067	0.977
		Illuminance	2	3.196	1.598	0.311	0.733
	RT ^a^	Temperature	2	4211.492	2105.746	4.544	0.011 *
		Noise	3	6441.957	2147.319	4.634	0.003 *
		Illuminance	2	122.755	61.378	0.132	0.876
	PI ^a^	Temperature	2	6.188	3.094	10.197	0.000 *
		Noise	3	2.042	0.675	2.224	0.085
		Illuminance	2	0.538	0.269	0.887	0.413

* Difference is significant at the *p* < 0.05 level (2-tailed). ^a^ AC—Accuracy Rate, RT—Reaction Time, PI—Performance Indicator.

**Table 4:** Interaction effects of indoor temperature, noise and illuminance on learning efficiency in different tasks.

			DF	SS	MS	F	*p*
Perception-oriented task	AC ^a^	Temperature × Noise	6	593.650	98.942	2.472	0.024 *
		Temperature × Illuminance	4	159.008	39.752	0.993	0.411
		Noise × Illuminance	6	115.778	19.296	0.482	0.822
		Temperature × Noise × Illuminance	12	556.346	46.362	1.158	0.312
	RT ^a^	Temperature × Noise	6	11,300.046	1883.341	2.155	0.047 *
		Temperature × Illuminance	4	1878.833	469.708	0.537	0.708
		Noise × Illuminance	6	13,754.610	2292.435	2.623	0.017 *
		Temperature × Noise × Illuminance	12	12,794.923	1066.244	1.220	0.268
	PI ^a^	Temperature × Noise	6	0.265	0.044	2.089	0.054
		Temperature × Illuminance	4	0.080	0.020	0.949	0.436
		Noise × Illuminance	6	0.300	0.050	2.370	0.030 *
		Temperature × Noise × Illuminance	12	0.387	0.032	1.528	0.113
Memory-oriented task	AC ^a^	Temperature × Noise	6	909.444	151.574	0.791	0.577
		Temperature × Illuminance	4	427.778	106.944	0.558	0.693
		Noise × Illuminance	6	312.778	52.130	0.272	0.950
		Temperature × Noise × Illuminance	12	352.222	29.352	0.153	1.000
	RT ^a^	Temperature × Noise	6	1714.152	285.692	4.703	0.000 *
		Temperature × Illuminance	4	905.583	226.396	3.727	0.006 *
		Noise × Illuminance	6	541.087	90.181	1.485	0.183
		Temperature × Noise × Illuminance	12	2307.325	192.277	3.165	0.000 *
	PI ^a^	Temperature × Noise	6	27.491	4.582	3.888	0.001 *
		Temperature × Illuminance	4	7.639	1.910	1.620	0.169
		Noise × Illuminance	6	10.209	1.702	1.444	0.197
		Temperature × Noise × Illuminance	12	39.091	3.258	2.764	0.001 *
Problem-solving-oriented task	AC ^a^	Temperature × Noise	6	328.889	54.815	0.136	0.991
		Temperature × Illuminance	4	917.778	229.444	0.570	0.684
		Noise × Illuminance	6	1628.889	271.481	0.675	0.670
		Temperature × Noise × Illuminance	12	3011.111	250.926	0.624	0.822
	RT ^a^	Temperature × Noise	6	29,068.415	4844.736	4.551	0.000 *
		Temperature × Illuminance	4	3051.334	762.834	0.717	0.581
		Noise × Illuminance	6	8474.340	1412.390	1.327	0.245
		Temperature × Noise × Illuminance	12	18,005.647	1500.471	1.409	0.160
	PI ^a^	Temperature × Noise	6	0.142	0.024	1.292	0.260
		Temperature × Illuminance	4	0.046	0.012	0.635	0.638
		Noise × Illuminance	6	0.141	0.024	1.291	0.261
		Temperature × Noise × Illuminance	12	0.370	0.031	1.687	0.068
Attention-oriented task	AC ^a^	Temperature × Noise	6	22.273	3.712	0.721	0.633
		Temperature × Illuminance	4	38.863	9.716	1.888	0.112
		Noise × Illuminance	6	15.226	2.538	0.493	0.813
		Temperature × Noise × Illuminance	12	46.285	3.857	0.750	0.702
	RT ^a^	Temperature × Noise	6	5646.301	941.050	2.031	0.061
		Temperature × Illuminance	4	1854.149	463.537	1.000	0.408
		Noise × Illuminance	6	7081.988	1180.331	2.547	0.020 *
		Temperature × Noise × Illuminance	12	10,469.745	872.479	1.883	0.036 *
	PI ^a^	Temperature × Noise	6	4.163	0.694	2.287	0.035 *
		Temperature × Illuminance	4	0.430	0.107	0.354	0.841
		Noise × Illuminance	6	3.988	0.665	2.191	0.044 *
		Temperature × Noise × Illuminance	12	5.196	0.433	1.427	0.152

* Difference is significant at the *p* < 0.05 level (2-tailed). ^a^ AC—Accuracy Rate, RT—Reaction Time, PI—Performance Indicator.

**Table 5:** Mean ± Standard Deviation (SD) for task PI (Performance Indicator) under different environmental scenarios.

Perception-oriented task		**17 °C**			**22 °C**			**27 °C**		
		**60 lx**	**300 lx**	**2200 lx**	**60 lx**	**300 lx**	**2200 lx**	**60 lx**	**300 lx**	**2200 lx**
	40 dB(A)	0.78 ± 0.12	0.66 ± 0.13	0.68 ± 0.12	0.65 ± 0.14	0.76 ± 0.13	0.79 ± 0.21	0.64 ± 0.17	0.63 ± 0.11	0.55 ± 0.12 ^b^
	50 dB(A)	0.62 ± 0.16	0.69 ± 0.11	0.75 ± 0.15	0.60 ± 0.12	0.67 ± 0.16	0.81 ± 0.21 ^a^	0.63 ± 0.21	0.63 ± 0.08	0.76 ± 0.13
	60 dB(A)	0.68 ± 0.18	0.64 ± 0.11	0.70 ± 0.13	0.70 ± 0.16	0.66 ± 0.13	0.72 ± 0.17	0.58 ± 0.06	0.64 ± 0.16	0.80 ± 0.16
	70 dB(A)	0.63 ± 0.13	0.60 ± 0.08	0.79 ± 0.16	0.56 ± 0.08	0.59 ± 0.20	0.64 ± 0.18	0.56 ± 0.10	0.62 ± 0.12	0.72 ± 0.16
Memory-oriented task		**17 °C**			**22 °C**			**27 °C**		
		**60 lx**	**300 lx**	**2200 lx**	**60 lx**	**300 lx**	**2200 lx**	**60 lx**	**300 lx**	**2200 lx**
	40 dB(A)	3.68 ± 1.09	2.77 ± 0.64	4.00 ± 1.91	3.38 ± 0.86	3.74 ± 1.10	3.83 ± 1.19	2.51 ± 0.80	3.61 ± 1.11	2.40 ± 0.64
	50 dB(A)	3.60 ± 1.25	3.39 ± 0.93	3.03 ± 1.04	2.68 ± 0.60	3.04 ± 1.27	4.04 ± 1.43	2.61 ± 0.94	4.08 ± 1.36 ^a^	4.07 ± 1.79
	60 dB(A)	3.40 ± 1.60	3.57 ± 1.19	2.88 ± 0.88	2.25 ± 0.42	3.08 ± 0.86	2.70 ± 0.89	3.11 ± 0.95	3.12 ± 1.20	3.50 ± 0.84
	70 dB(A)	2.78 ± 0.71	3.06 ± 1.16	3.96 ± 1.83	2.17 ± 0.60 ^b^	2.28 ± 0.50	2.22 ± 0.56	2.50 ± 0.73	2.41 ± 0.84	3.79 ± 1.09
Problem-solving-oriented task		**17 °C**			**22 °C**			**27 °C**		
		**60 lx**	**300 lx**	**2200 lx**	**60 lx**	**300 lx**	**2200 lx**	**60 lx**	**300 lx**	**2200 lx**
	40 dB(A)	0.51 ± 0.13	0.39 ± 0.10	0.50 ± 0.21	0.46 ± 0.11	0.52 ± 0.20 ^a^	0.46 ± 0.17	0.41 ± 0.11	0.48 ± 0.14	0.38 ± 0.15
	50 dB(A)	0.42 ± 0.13	0.47 ± 0.11	0.47 ± 0.17	0.44 ± 0.13	0.40 ± 0.10	0.49 ± 0.16	0.39 ± 0.15	0.49 ± 0.13	0.47 ± 0.19
	60 dB(A)	0.38 ± 0.12	0.42 ± 0.11	0.36 ± 0.14	0.32 ± 0.14	0.44 ± 0.14	0.33 ± 0.15	0.32 ± 0.11	0.38 ± 0.09	0.49 ± 0.14
	70 dB(A)	0.36 ± 0.10	0.39 ± 0.09	0.47 ± 0.13	0.30 ± 0.11 ^b^	0.33 ± 0.13	0.35 ± 0.14	0.38 ± 0.13	0.32 ± 0.08	0.45 ± 0.13
Attention-oriented task		**17 °C**			**22 °C**			**27 °C**		
		**60 lx**	**300 lx**	**2200 lx**	**60 lx**	**300 lx**	**2200 lx**	**60 lx**	**300 lx**	**2200 lx**
	40 dB(A)	1.69 ± 0.69	1.38 ± 0.42	2.13 ± 1.02 ^a^	1.50 ± 0.28	1.65 ± 0.65	1.68 ± 0.57	1.30 ± 0.19	1.37 ± 0.24	1.34 ± 0.37
	50 dB(A)	1.42 ± 0.39	1.62 ± 0.60	1.72 ± 1.11	1.21 ± 0.22	1.26 ± 0.27	1.59 ± 2.37	1.68 ± 0.84	1.50 ± 0.31	1.51 ± 0.26
	60 dB(A)	1.77 ± 0.94	1.74 ± 0.51	1.03 ± 0.27 ^b^	1.22 ± 0.38	1.40 ± 0.37	1.17 ± 0.25	1.40 ± 0.48	1.39 ± 0.39	1.42 ± 0.25
	70 dB(A)	1.71 ± 0.98	1.52 ± 0.41	1.95 ± 1.38	1.18 ± 0.17	1.06 ± 0.12	1.11 ± 0.13	1.40 ± 0.38	1.06 ± 0.19	1.43 ± 0.23

^a^ The highest PI achieved in each type of task. ^b^ The lowest PI achieved in each type of task.
